# Sclerostin as a new target of diabetes-induced osteoporosis

**DOI:** 10.3389/fendo.2024.1491066

**Published:** 2024-12-10

**Authors:** Yanhua Li, Yaheng Luo, Debin Huang, Lele Peng

**Affiliations:** ^1^ Department of Endocrinology and Metabolism, The Third Hospital of Changsha, Changsha, Hunan, China; ^2^ Department of Endocrinology and Metabolism, Want Want Hospital, Changsha, Hunan, China

**Keywords:** sclerostin, type 1 diabetes, type 2 diabetes, osteoporosis, pathogenesis, target

## Abstract

Sclerostin, a protein synthesized by bone cells, is a product of the *SOST* gene. Sclerostin is a potent soluble inhibitor of the WNT signaling pathway, and is known to inhibit bone formation by inhibiting osteocyte differentiation and function. Currently, sclerostin has been the subject of numerous animal experiments and clinical investigations. By conducting a literature review, we have gained insights into the most recent advancements in research. Patients with both type 1 diabetes and type 2 diabetes have high levels of serum sclerostin. Patients with type 1 diabetes and type 2 diabetes are both more likely to suffer from osteoporosis, and serum sclerostin levels are elevated in osteoporosis. Many studies have confirmed that sclerostin has been implicated in the pathogenesis of osteoporosis, so we speculate that sclerostin plays an important role in osteoporosis through the glucose metabolism pathway, which may promote the osteoporosis of morbidity in type 1 diabetes and type 2 diabetes. Based on this, we propose whether serum sclerostin can predict type 1 diabetes and type 2 diabetes-induced osteoporosis, and whether it can be a new target for the prevention and treatment of type 1 diabetes and type 2 diabetes-induced osteoporosis, providing new ideas for clinicians and researchers.

## 1 Introduction

The prevalence of diabetes is escalating, making it a significant chronic epidemic. The International Diabetes Federation reports that approximately 537 million individuals worldwide are currently afflicted with diabetes, constituting 10.5% of the global population (1). The prevalence of diabetes is projected to increase to 783 million individuals by 2045 (1). Another metabolic disease with increasing prevalence is osteoporosis. Osteoporosis, marked by reduced bone density, alterations in bone microstructure, and subsequent fractures, leads to substantial disability and mortality, and has now become a global health concern (2, 3). According to the International Osteoporosis Foundation, with the global population aging, it is projected that over 200 million individuals currently endure the condition of osteoporosis, and one in three women and one in five men over the age of 50 will experience an osteoporotic fracture (4).

Through literature review and previous research, we have learned that sclerostin plays an important role in the pathogenesis of osteoporosis, especially when accompanied by abnormal glucose metabolism. Based on this, this article reviews the clinical evidence regarding serum sclerostin in diabetes and osteoporosis, and delve into the underlying mechanisms involved. This will provide a basis for sclerostin as a new biomarker for diabetes-induced osteoporosis, as well as for potential therapeutic targets. It also provides reference for further clinical research and scientific basis for new drug development.

## 2 Structure, expression, functions, and signaling pathway related to sclerostin

Sclerostin is a secreted glycoprotein composed of 213 amino acid residues, originally derived from high bone mass disorders sclerosis and van Buchem’s disease (5, 6). Sclerostin is a 22-kDa protein characterized by a core disulfide-bonded structure composed of three distinct domains: ring 1, ring 2, and ring 3 (7); the protein’s side chain features a highly flexible N-terminal domain (amino acids 1-55) and a C-terminal domain (amino acids 145-189); it contains four disulfide bonds formed by four p-cysteine residues (8). Unlike the majority of proteins containing cystine junction motifs, sclerostin exists as a monomer (9). Sclerostin is synthesized through the expression of the *SOST* gene on human chromosome 17q12-q21 (10). The *SOST* gene contains two distinct transcription sites. The first transcription site is evolutionary conserve region 5, and monocyte enhancer factor 2 can promote the expression of sclerostin by binding to evolutionary conserve region 5 (11, 12). However, histone deacetylases 4 and 5 are capable of inhibiting the transcription of the *SOST* gene by binding to monocyte enhancer factor 2 (13). The second transcription site is the upstream promoter region, where runt-related transcription factor 2 binds and represses sclerostin expression (14, 15). Histone deacetylases 3 inhibits the transcription of the *SOST* gene by targeting the promoter region (16, 17). Sclerostin is mainly secreted by osteocytes and acts in a paracrine manner (18). It is detectable in plasma and expressed in tissues such as bone, cartilage, kidney, liver, pancreas, heart and blood vessels (19). Sclerostin expression is markedly reduced in newly embedded osteocytes and undetectable in mature osteoblasts and bone lining cells (20). Sclerostin, an inhibitor of the WNT signaling pathway, antagonizes bone formation by binding to low-density lipoprotein receptor-related protein (LRP) 5/6, functioning as an antianabolic agent (21, 22). It is also implicated in skeletal muscle regeneration, insulin resistance, and glucose metabolism (23, 24). Recent investigations have demonstrated that sclerostin potentiates the inhibitory effect on bone formation by mediating binding to LRP6 through its interaction with LRP4 (25).

## 3 Expression of sclerostin in diabetes

In recent years, the incidence of type 1 diabetes (T1D) has increased rapidly at a rate of 3%-5% per year worldwide. Wedrychowicz, et al. (26) demonstrated that serum sclerostin levels are significantly elevated in patients with T1D and exhibit an inverse correlation with glycosylated hemoglobin (HbA1c). Rubin et al. (27) conducted a cross-sectional study, which found that sclerostin was significantly increased in T1D patients; However, sclerostin is not associated with HbA1c. This may be because the participants are older, and the level of sclerostin increases with age, with higher values masking the relationship with HbA1c. Neumann et al. (28) conducted a study on T1D and healthy individuals, found that the level of sclerostin in T1D was significantly higher than that in the control group, and was not associated with bone metabolism markers. Therefore, we speculate that sclerostin may be involved in osteoporosis independently of bone metabolism markers. Kurban et al. (29) conducted a cross-sectional study comparing the levels of sclerostin between 40 T1D and 40 healthy controls, and found that sclerostin was elevated in T1D, but the difference was not statistically significant, possibly due to the small sample size. Employing recent research, it has been demonstrated that the serum level of sclerostin is markedly elevated in patients with T1D in comparison to healthy controls (30). Faienza’s research also confirmed the same results (31).

A lot of studies have also been conducted on the expression of sclerostin in type 2 diabetes (T2D). Sclerostin levels were reported to be higher in patients with T2D in an age-matched randomized controlled study (32). Garcia-Martin et al. (33) conducted a cross-sectional study, revealing that sclerostin levels are elevated in patients with T2D and demonstrating a correlation between sclerostin levels and the duration of T2D, HbA1c. The findings of a clinical investigation conducted by Singh et al. (34), involving a cohort of 171 individuals divided into three categories—healthy individuals, individuals with pre-T2D, and patients with T2D—demonstrated a gradual increase in sclerostin levels and sclerostin mRNA expression from healthy to pre-T2D to T2D. In addition, elevated circulating sclerostin levels were positively correlated with insulin resistance and fat mass (35). Frysz et al. (36) conducted a meta-analysis, revealing a significant association between elevated levels of sclerostin and an increased risk of diabetes. A cross-sectional analysis of femoral head bone tissue from postmenopausal women with T2D revealed a significant increase in the expression of *SOST*, when compared to healthy women (37) (**Table 1**).

**Table 1:** Changes of sclerostin in patients with T1D and T2D.

Authors,Year	Type of Study	Study Subjects	Major Findings
Type 1 diabetes			
Wedrychowicz et al., 2019 (26)	A cross-sectional study	40 patients with T1D and 28 healthy as controls	Sclerostin levels were significantly higher in patients with T1D than in the control group without significant differences between genders
Rubin et al., 2022 (27)	A cross-sectional study	232 T1D participants and 104 control participants without diabetes followed for >30 years	Compared with the control participants, T1D had higher levels of sclerostin
Neumann et al., 2014 (28)	A cross-sectional study	128 men and premenopausal women with long-standing T1D and 77 age-, body mass index and gender-matched healthy individuals	Serum sclerostin levels were increased in patients with T1D, and the positive correlation of age with serum sclerostin levels was stronger in T1D
Kurban et al., 2022 (29)	A cross-sectional study	40 children and adolescents with T1D between the ages of 7 and 17, and 40 healthy children and adolescents between the ages of 6 and 17	The level of sclerostin in T1D is elevated, but the difference is not statistically significant, which may be due to the small sample size
Hygum et al., 2017 (30)	A systematic review and meta-analysis	–	Serum sclerostin was significantly higher in patients with T1D compared with controls
Faienza et al., 2017 (31)	A cross-sectional study	106 T1D subjects and 80 controls	Serum sclerostin levels are elevated in patients with T1D
Type 2 diabetes			
Gennari et al., 2012 (32)	A cross-sectional study	40 T2D and 43 T1D patients were studied and compared with a reference control group (n = 83)	Sclerostin levels were higher in T2D than in controls or T1D patients
Garcia-Martin et al., 2012 (33)	A cross-sectional study	T2D group (n = 74) and control group (n = 50)	Sclerostin levels were significantly higher in T2D patients than control subjects and in T2D males than in T2D females
Singh et al., 2022 (34)	An observational study	A total of 171 study participants were enrolled in T2D, pre-T2D, and controls groups, having 57 each in the group	From healthy to pre-T2D to T2D, the level of sclerostin increased gradually
Frysz et al., 2022 (36)	A meta-analysis	5069 participants with complete data	Higher sclerostin levels were associated with higher risk of T2D, risk of elevated fasting glucose, and triglyceride levels
Piccoli et al., 2020 (37)	A cross-sectional study	Bone tissue from femoral heads of 19 T2D postmenopausal women and 73 age- and body mass index-comparable nondiabetic women undergoing hip replacement surgery	A significantly higher *SOST* (p = 0.006) in T2D compared with non-diabetic subjects

Therefore, we speculate that sclerostin is not only involved in the pathogenesis of diabetes, but also plays an important role in osteoporosis through glucose metabolism.

## 4 Association of sclerostin with osteoporosis in diabetes

### 4.1 Association of diabetes with osteoporosis

Diabetes is considered a significant risk factor for osteoporosis, as an increasing body of evidence supports its association with an elevated risk of osteoporotic fractures (38–41). Glycolysis can promote osteoblast differentiation, may alter the levels of important intermediate metabolites that regulate gene expression (42). In their review, Vadivalagan et al. identified aerobic glycolysis as an effective way to accelerate the treatment of osteoporosis (43). Poor blood sugar control is an important risk factor for diabetic osteoporosis fracture (44). Nirwan and Vohora (45) performed experiments in *C57BL/6* mice, which were fed a high-fat diet for 22 weeks to induce diabetic osteoporosis. Subsequently, linagliptin combined with metformin was used for intervention, and the results suggested that diabetic osteoporosis could be treated by increasing the level of bone morphogenetic protein-2 and reducing the level of sclerostin.

Previous studies have confirmed that T1D is closely related to decreased bone mineral density (BMD) and bone quality (46, 47). Studies have reported that children and adolescents with T1D compared with healthy controls, the BMD value is low (48), lead to adult peak bone mass was lower than those of healthy people, thus prone to osteopenia and osteoporosis (49). The longer the duration of T1D and the worse the blood sugar control, the higher the risk of fractures in patients (46, 47, 50). Two previous cross-sectional studies have demonstrated that poorer glycemic control was associated with lower BMD scores in patients with T1D (51). This is due to reduced bone production and formation of mineralized matrix in T1D (52, 53). It is known that T1D has a negative effect on osteoblast differentiation and function and a positive effect on osteoclast differentiation and function, thereby reducing bone formation and increasing bone resorption (54). The reason may be that hyperglycemia inhibits osteogenic differentiation of mesenchymal stem cells (55), and inhibits the ability of osteoblasts to resist mechanical load (56, 57). It has been established that the bone phenotype of patients with T1D is characterized by the following four characteristics: reduced BMD (58), disrupted bone microstructure (59), increased risk of fractures (60), reduced the conversion rate of bone (61). And risk of fracture in patients with T1D is six times that of the healthy adults (62). A meta-analysis of 46 studies with 2617 and 3851 controls showed that children with T1D had significantly lower BMD measured by dual-energy X-ray absorptiometry (whole body, lumbar spine, femur), peripheral bone quantitative CT scanning (radius and tibia), and quantitative ultrasound of calcaneus and phalanges compared with controls (63). The findings from the study conducted by Weber et al. (64) demonstrated a significant reduction in bone mass gain among individuals diagnosed with T1D one year after diagnosis. Kalaitzoglou et al. (65) used streptozotocin to induce T1D in mice. The results suggest that chronic hyperglycemia and pro-inflammatory bone microenvironment in T1D mice enhance osteoclast activity, which leads to enhanced bone resorption and decreased bone mass.

A state of low bone turnover has been demonstrated in T2D (66–68). Hygum et al. (30) conducted a meta-analysis and found that levels of bone formation markers (N-terminal propeptide of type 1 procollagen (P1NP) and osteocalcin) and bone resorption markers (C-terminal cross-linked telopeptide of type I collagen (CTX) and tartrate-resistant acid phosphatase 5b isoform) were reduced in T2D. The study by Napoli et al. (69) showed that P1NP was reduced by about 13% and CTX was reduced by about 43% in patients with T2D compared with non-diabetic subjects. It has also been confirmed that the risk of fragility fractures is increased in patients with T2D (33, 70). Strotmeyer et al. (71) conducted a cohort study, results show that the adult T2D patients than about 64% higher risk of fracture in patients without T2D.

Both types of diabetes are susceptible to osteoporosis, suggesting that the two forms of diabetes induce the development of osteoporosis through different mechanisms (72). Insufficient osteoblast differentiation is considered to be an important cause of osteoporosis in T1D patients (73); The inhibition of bone remodeling is considered a significant contributing factor of osteoporosis in T2D patients (74). However, the specific pathogenesis of osteoporosis in diabetes is not clear.

### 4.2 Expression of sclerostin in diabetes-induced osteoporosis

Recent studies have shown that sclerostin is involved in bone metabolism in T1D (31). Clinical data from Wędrychowicz et al. (26) could point to increased sclerostin levels as a potential cause of reduced bone formation in T1D. Yee et al. (75) conducted an animal study to establish a streptozotocin-induced fracture model of T1D mice. The researchers injected sclerostin antibody into the mouse model, and on days 21 and 42, a large number of early osteoblasts were seen, and bone quality was significantly improved.

The same changes were also observed in T2D. Yamamoto’s study not only confirmed the elevation of sclerostin in T2D, but also revealed that sclerostin increases vertebral fractures, which may be due to sclerostin mediated deterioration of bone quality (76). Ardawi et al. (77) conducted a cross-sectional study on 482 T2D patients and 482 healthy individuals, and the results showed that sclerostin was elevated in T2D, and sclerostin was associated with increased bone fragility. Wang et al. (78) included 95 T2D patients and divided them into normal bone group, osteopenia group and osteoporosis group according to bone mineral density, and found that the osteoporosis group had the highest level of sclerostin. The researchers propose that sclerostin mediates osteoporosis in T2D by inhibiting WNT signaling. In a cross-sectional study conducted by Ahmad (79), we learned that sclerostin was significantly higher in T2D compared to healthy people; Moreover, the incidence of osteopenia and osteoporosis in T2D is higher than that in the healthy people. Animal study by Hamann et al. (80) concluded that sclerostin antibody therapy reversed the adverse effects of T2D on bone mass and strength in rats and improved bone defect regeneration, suggesting that sclerostin could be used as a biomarker for early detection of osteoporosis in diabetes patients.

From the above studies, we learned that sclerostin may be involved in the pathogenesis of osteoporosis through glucose metabolism.

## 5 Relationship between sclerostin and clinical outcome in osteoporosis

Owing to its recognized role as a negative regulator of the WNT signaling pathway, sclerostin binds to LRP5/6 coreceptors, thereby inhibiting bone formation and promoting bone resorption (81). It has also been shown that sclerostin stimulates bone resorption through receptor activator of nuclear factor kappa-B ligand-dependent pathway, thereby promoting BMD reduction (82). Sclerostin has been demonstrated to suppress the proliferation of osteoblasts while simultaneously promoting their apoptosis (83). Cosman et al. (84) performed a randomized, controlled clinical trial on postmenopausal women with osteoporosis and demonstrated that treatment with anti-sclerostin antibodies enhances bone formation and increases BMD, thereby mitigating the risk of fracture. A 12-month phase IIb trial, involving postmenopausal women randomized to receive either teriparatide (20ug/day) or anti-sclerostin antibody (210mg/month), demonstrated a significant increase in lumbar BMD with the administration of anti-sclerostin antibody therapy (85). In a meta-analysis published in 2022 (86), Poutoglidou et al. observed that both a 6-month and a 12-month treatment course with anti-sclerostin antibodies were capable of enhancing BMD at the lumbar spine, total hip, and femoral neck, and lowering the incidence of fractures. Concurrently, anti-sclerostin antibody therapy also demonstrated a reduction in CTX levels and an elevation in P1NP levels. Therefore, the usage of anti-sclerostin monoclonal antibodies has progressively become a significant component in the management of osteoporosis (87). In a phase III clinical trial, the subcutaneous administration of antibodies to sclerostin (such as romosozumab, a monoclonal antibody that binds sclerostin) demonstrated efficacy in enhancing BMD compared to the placebo in postmenopausal women with osteoporosis and men with osteoporosis (84, 88). The findings of Recker et al. (89), Cosman et al. (84) and Kaveh et al. (90) corroborated this observation. A case report suggests that the nonunion of humeral shaft fractures in males and postmenopausal females might be addressed through the administration of anti-sclerostin medications (91) (**Table 2**).

**Table 2:** Changes in the serum sclerostin levels in osteoporosis.

Authors,Year	Type of Study	Study Subjects	Major Findings
Cosman et al., 2016 (84)	A Randomized controlled clinical trial	7180 postmenopausal women who had a T score of -2.5 to -3.5 at the total hip or femoral neck	Anti-sclerostin antibody therapy can increase bone formation and increase BMD, thereby reducing the risk of fracture
McClung et al., 2014 (85)	A Phase II, multicenter, international, randomized, placebo-controlled, parallelgroup, eight-group study	419 postmenopausal women	Anti-sclerostin antibody therapy can significantly improve BMD at the lumbar spine
Poutoglidou et al., 2022 (86)	A Meta-Analysis and Systematic Review	–	At 6 and 12 months, anti-sclerostin antibody significantly increase BMD in the lumbar spine, total hip and femoral neck
Lewiecki et al., 2018 (88)	A Phase III Randomized Placebo-Controlled Trial	245 subjects (163 romosozumab, 82 placebo)	Anti-sclerostin antibody therapy can improve BMD in male patients with osteoporosis
Recker et al., 2015 (89)	A Randomized, Double‐Blind Phase 2 Clinical Trial	120 postmenopausal women between 45 and 85 years of age, with a lumbar spine BMD T‐score of –2.0 to –3.5, inclusive	Anti-sclerostin antibody treatment can significantly increase BMD in the spine, femoral neck, and total hip as compared with placebo, which is dose-dependent
Kaveh et al., 2020 (90)	A Systematic review and Meta-analysis	–	Treatment with anti-sclerostin antibody can be a proper therapeutic option in patients with osteoporosis and low BMD
Lee et al., 2022 (91)	A case report	a 67-year-old woman with nonunion of humerus shaft fracture	Anti-sclerostin antibody therapy can aid in promoting bone healing of nonunion

Enhanced bone formation has been observed in patients or mice lacking sclerostin, leading to bone sclerosis, as demonstrated by the results of several studies (10, 92, 93); In contrast, mice with an excessive expression of *SOST* exhibited reduced bone mass (94). *In vitro* experiments by Wang et al. (95) revealed that overexpression of *SOST* significantly inhibited WNT signaling and messenger ribonucleic acid levels of osteogenic markers in *Col1a2^+/G610C^* mouse osteoblasts. The domain in which sclerostin plays a major role in this process is loop 3. The elimination of *SOST* led to an increase in bone mass and strength (96, 97). In the mouse studies conducted by Oh et al. (98), schnurri-3 was observed to suppress *SOST* expression in osteoblasts, while the absence of *SOST* had no impact on schnurri-3. Specifically, targeted inhibition of both schnurri-3 and *SOST* effectively mitigated bone loss and stimulated bone formation in mice. Li et al. (81) developed a male rat model of osteoporosis and demonstrated that anti-sclerostin antibodies could significantly enhance bone mass and preserve bone quality in rats, primarily by stimulating bone formation and inhibiting bone resorption. The study conducted by Boyce et al. (99) demonstrated that administration of anti-sclerostin antibodies enhanced BMD and elevated serum concentrations of bone formation markers, but failed to affect levels of bone resorption markers. Brent et al. (100) employed a rat model, utilizing either anti-sclerostin antibody, abaloparatide, or a combination of both, and demonstrated that anti-sclerostin antibody enhanced bone strength in the mid-diaphysis, neck, and metaphysis of long bones. In contrast, the combination of anti-sclerostin antibody and abaloparatide led to a significant increase in bone strength at all sites, augmented markers of bone remodeling, and reduced trabecular bone spacing. This suggests that the combined treatment is significantly more effective than either agent alone. In a mouse model of streptozotocin-induced T1D, osteoblastic defects and reduced levels of osteocalcin and alkaline phosphatase were found to be associated with increased expression of WNT signaling inhibitors Dickkopf-1 and *SOST* (101). Interestingly, treatment with anti-sclerostin antibodies accelerates fracture healing by facilitating osteoblast differentiation and enhancing callus mineralization, thereby ameliorating bone microstructure (75). Maillard et al. (102) artificially induced skull defects in mice, and subsequently employed mesenchymal stem cells to counteract sclerostin. Eight weeks post-intervention, it was discovered that this approach not only increased bone formation and promoted bone repair, but also demonstrated comparable efficacy to *SOST* knockout mice. Hamann et al. (80) and Kruck et al. (103), respectively, demonstrated in animal models that the administration of anti-sclerostin antibodies accelerates bone formation. We are informed that anti-sclerostin antibodies act as an stimulator of bone formation in the short term and as an inhibitor of bone resorption in the long term, jointly leading to an increase in bone mass by both means (104). In a study employing *SOST* knockout mice, we observed that glucocorticoid administration led to a reduction in osteoprotegerin levels and an increase in the receptor activator of nuclear factor kappa-B ligand/osteoprotegerin ratio; Conversely, administration of an anti-sclerostin antibody inhibited bone resorption by augmenting osteoprotegerin levels (105). The research conducted by Lin et al. (106) demonstrated that mice lacking sclerostin exhibited resistance to bone mass decline. Carro Vazquez at el (107). demonstrated that treatment with anti-sclerostin antibodies could enhance bone quality and facilitate bone healing in rats, using the Zucker Diabetic Fatty rat model. The mechanism involves directly influencing bone by down-regulating miR-145-5p/p transcription in bone tissue, up-regulating osteoprotegerin target expression, resulting in decreased osteoclast production (108), and up-regulating Sp7 (109) and signal transduction 3A target levels (110), leading to enhanced osteogenic differentiation. The expression of the *SOST* gene and protein was suppressed by mechanical loading, leading to an enhancement in bone formation (106, 111, 112). The mechanism may be that mechanical stimulation activates connexin 43 hemichannells to release prostaglandin E2 from osteocytes, thereby inhibiting the expression of *SOST* in osteocytes and enhancing the activity of osteoblasts and bone formation (113). The research findings by Kim et al. (114) demonstrate that *SOST*-/- mice exhibit enhanced bone formation and reduced visceral and subcutaneous fat deposition, primarily attributed to the absence of sclerostin protein which inhibits the differentiation of progenitor cells into mature adipocytes. Thus inhibit sclerostin can also help the treatment of obesity. In Zhou et al.’s study (115), 23-month-old male rats were randomly divided into a orchiectomy group and a sham operation group. Eight weeks after surgery, the results showed that the serum sclerostin level in the orchiectomy group was significantly higher than that in the sham operation group, and was negatively correlated with trabecular BMD. This also suggests that sclerostin may be a potential therapeutic target for male osteoporosis. In both animal models and clinical studies, sclerostin antibody-induced bone formation was reactivated upon exposure to physical stimuli (84, 116, 117). Many non clinical pharmacology research results show that the sclerostin antibody can inhibit sclerostin to form, which can promote the fracture healing and callus formation (118). The mechanism of action is that sclerostin antibody inhibits the binding of sclerostin to LRP5/6, thereby weakening the antagonistic activity of sclerostin against WNT-induced responses (119).

Taking the above findings together, sclerostin is emerging as a potent inhibitor of bone formation by reducing osteoblast differentiation and activity. As a result, we speculate that sclerostin can be used as a biomarker for osteoporosis.

## 6 Conclusion and prospects

**Figure 1:**
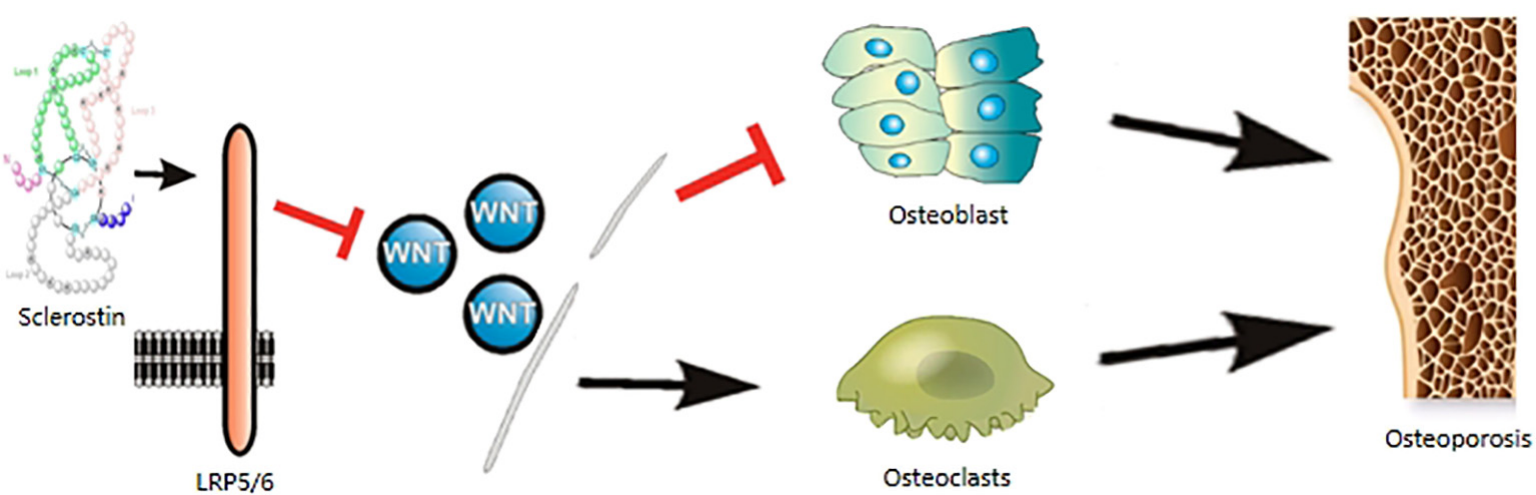
Sclerostin are involved in the mechanism of osteoporosis. Sclerostin binds to LRP5/6 coreceptors, acts on the WNT signaling pathway, inhibits osteoblasts and promotes osteoclastogenesis, leading to osteoporosis.

Sclerostin is a powerful protein molecules involved in bone metabolism and skeletal muscle regeneration, mainly related to osteoporosis (**Figure 1**). A large number of studies have revealed that sclerostin is elevated in T1D and T2D, and diabetic patients are more susceptible to osteoporosis. Numerous clinical studies have also demonstrated that patients with osteoporosis exhibit elevated levels of serum sclerostin. Existing evidence suggests that sclerostin antibodies such as romosozumab reduce sclerostin expression, leading to improvement in osteoporosis. Therefore, early exploration of sclerostin targets in diabetes patients plays a vital role in the prevention and treatment of diabetes-induced osteoporosis. However, sclerostin can be used in clinical medicine is still to be solved.

To fill the current gap, the following research is needed: To further clarify the specific mechanism of sclerostin in diabetes-induced osteoporosis, remove obstacles for clinical study; To develop safe and effective sclerostin inhibitors, prevent and treat osteoporosis induced by diabetes is warranted.
